# Walking and Other Common Physical Activities Among Adults with Arthritis — United States, 2019

**DOI:** 10.15585/mmwr.mm7040a3

**Published:** 2021-10-08

**Authors:** Dana Guglielmo, Louise B. Murphy, Kristina A. Theis, Michael A. Boring, Charles G. Helmick, Kathleen B. Watson, Lindsey M. Duca, Erica L. Odom, Yong Liu, Janet B. Croft

**Affiliations:** ^1^Division of Population Health, National Center for Chronic Disease Prevention and Health Promotion, CDC; ^2^Oak Ridge Institute for Science and Education, Oak Ridge, Tennessee; ^3^Division of Nutrition, Physical Activity, and Obesity, CDC; ^4^Epidemic Intelligence Service, CDC, National Center for Chronic Disease Prevention and Health Promotion, CDC.

The numerous health benefits of physical activity include reduced risk for chronic disease and improved mental health and quality of life (*1*). Physical activity can improve physical function and reduce pain and fall risk among adults with arthritis, a group of approximately 100 conditions affecting joints and surrounding tissues (most commonly osteoarthritis, fibromyalgia, gout, rheumatoid arthritis, and lupus) (*1*). Despite these benefits, the 54.6 million U.S. adults currently living with arthritis are generally less active than adults without arthritis, and only 36.2% of adults with arthritis are aerobically active (i.e., meet aerobic physical activity guidelines*) (*2*). Little is known about which physical activities adults with arthritis engage in. CDC analyzed 2019 Behavioral Risk Factor Surveillance System (BRFSS) data to examine the most common nonwork–related physical activities among adults with arthritis who reported any physical activity during the past month, nationally and by state. In 2019, 67.2% of adults with arthritis reported engaging in physical activity in the past month; among these persons, the most commonly reported activities were walking (70.8%), gardening (13.3%), and weightlifting (7.3%). In 45 U.S. states, at least two thirds of adults with arthritis who engaged in physical activity reported walking. Health care providers can help inactive adults with arthritis become active and, by encouraging physical activity and referring these persons to evidence-based physical activity programs, improve their health and quality of life.

BRFSS is an ongoing, state-based landline and cellular telephone survey of noninstitutionalized U.S. adults aged ≥18 years conducted by health departments in 50 states, the District of Columbia (DC), and U.S. territories.^†^ In 2019, the median response rate among the 49 states included in this analysis^§^ was 49.4% (range = 37.3%–73.1%).^¶^ Arthritis was defined as an affirmative response to the question, “Have you ever been told by a doctor or other health care professional that you have arthritis, rheumatoid arthritis, gout, lupus, or fibromyalgia?”** Engaging in physical activity was defined as responding “yes” to the question, “During the past month, other than your regular job, did you participate in any physical activities or exercises such as running, calisthenics, golf, gardening, or walking for exercise?” Among the 380,418 (92.8%) BRFSS respondents in 49 states and DC who reported arthritis status, age, and physical activity status, 87,299 (22.9%) reported having arthritis and engaging in physical activity. These participants were asked to report up to two activities in which they most frequently engaged from a list of 74 activities.^††^

Unadjusted percentages for each activity were calculated for the combined 49 states and DC. Age-specific and age-adjusted^§§^ percentages for the three most commonly reported activities (walking, gardening, and weightlifting) were calculated for adults with arthritis engaging in nonwork–related physical activity by selected sociodemographic and health-related characteristics, including joint pain severity, body mass index, physical limitations, and self-rated health. Unadjusted state-specific prevalences of walking, gardening, and weightlifting among adults with arthritis were also estimated. Paired t-tests were performed to assess differences across subgroups for all variables, and linear trend tests using orthogonal linear contrasts were conducted for ordinal variables; all comparisons reported are statistically significant (p-value <0.05). Analyses accounted for BRFSS’s complex sampling design, were weighted to be representative of each state, and were conducted using SAS (version 9.4; SAS Institute) and SUDAAN (version 11.0; RTI International). This activity was reviewed by CDC and was conducted consistent with applicable federal law and CDC policy.^¶¶^

In 2019, 67.2% of adults with arthritis engaged in nonwork–related physical activity in the past month; walking was the most commonly reported activity (70.8%), followed by gardening (13.3%), and weightlifting (7.3%) (Table 1). The percentage reporting walking was lowest among those 18–44 years (63.7%) (Table 2). The age-adjusted prevalence of walking was higher among women (76.0%) than among men (63.9%), higher among non-Hispanic Black (75.4%) adults than among non-Hispanic White (70.0%) and non-Hispanic other/multiple race adults (68.3%), and higher among those who were unable to work or disabled (79.0%) compared with those adults with other employment statuses (67.7%–74.8%). The age-adjusted percentage of adults with arthritis who reported walking increased with increasing joint pain severity and body mass index, and decreased with increasing education, income, and self-rated health.

**TABLE 1:** Weighted unadjusted percentages of adults with arthritis* who reported engaging in physical activity in the past month,^†^ reporting first or second most frequent activities^§^ — Behavioral Risk Factor Surveillance System, United States,^¶^ 2019

Activity group**	No. of respondents	% (95% CI)
**Walking or backpacking**	**62,902**	**72.1 (71.4–72.7)**
Walking	61,931	70.8 (70.2–71.4)
Hiking or backpacking	1,312	1.6 (1.5–1.8)
**Lawn and garden**	**18,297**	**19.6 (19.1–20.2)**
Gardening	12,094	13.3 (12.8–13.8)
Yard work	6,585	6.6 (6.3–7.0)
**Muscle strengthening**	**9,885**	**12.8 (12.3–13.2)**
Weightlifting	5,357	7.3 (7.0–7.7)
Calisthenics^††^	2,014	2.6 (2.4–2.8)
Yoga	2,368	2.7 (2.5–2.9)
Pilates	349	0.4 (0.3–0.5)
**Aerobic conditioning exercise**	**9,196**	**10.0 (9.6–10.4)**
Bicycling machine exercise	4,241	4.5 (4.2–4.8)
Aerobics video or class	2,210	2.4 (2.2–2.6)
Elliptical or elliptical fitness crosstrainer machine exercise	1,675	2.1 (1.9–2.3)
Stair climbing or StairMaster	959	0.9 (0.8–1.1)
Other aerobic conditioning exercise	377	0.4 (0.4–0.5)
**Home activities^§§^**	**7,621**	**7.9 (7.5–8.2)**
**Sports**	**5,115**	**6.3 (6.0–6.7)**
Golf	2,571	2.9 (2.7–3.1)
Bowling	394	0.5 (0.4–0.6)
Tennis	379	0.5 (0.4–0.6)
Other sports	1,881	2.6 (2.4–2.9)
**Running or jogging**	**2,459**	**4.5 (4.2–4.9)**
**Water activities**	**3,654**	**4.4 (4.2–4.7)**
Swimming	3,345	4.1 (3.8–4.4)
Other water activities	315	0.3 (0.3–0.4)
**Bicycling**	**3,314**	**4.3 (4.0–4.6)**
**Dancing**	**966**	**1.3 (1.2–1.5)**
**Fishing and hunting**	**716**	**0.9 (0.8–1.0)**
**Farm or ranch work**	**1,182**	**0.9 (0.8–1.0)**
**Winter activities**	**900**	**0.6 (0.5–0.7)**
Snow shoveling by hand	626	0.4 (0.4–0.5)
Other winter activities	286	0.2 (0.1–0.2)

**Abbreviations:** BRFSS = Behavioral Risk Factor Surveillance System; CI = confidence interval.

* Respondents were classified as having arthritis if they responded “Yes” to the question, “Have you ever been told you have some form of arthritis, rheumatoid arthritis, gout, lupus, or fibromyalgia?”

^†^ Respondents with arthritis were classified as engaging in physical activity if they responded “Yes” to the question, “During the past month, other than your regular job, did you participate in any physical activities or exercises such as running, calisthenics, golf, gardening, or walking for exercise?”

^§^ Those who engaged in physical activity were classified as participating in an activity if they reported this activity in response to two questions: 1) “What type of physical activity or exercise did you spend the most time doing during the past month?” or 2) “What other type of physical activity gave you the next most exercise during the past month?” Participants who reported one activity but had missing data for the other most frequent activity (e.g., “don’t know” or “refused”) were included in the analysis. The sum of respondents for all activities exceeds the total number of respondents since each respondent could report up to two activities. Survey interviewers coded activities not listed among the 74 activities in the BRFSS Activity List for Common Leisure Activities into a single, heterogeneous “other” category representing a wide variety of different activities (n = 13,241; 13.7% [95% CI: 13.2–14.1]). https://www.cdc.gov/brfss/annual_data/2019/pdf/codebook19_llcp-v2-508.HTML

^¶^ In 2019, New Jersey did not collect enough data to meet the minimum requirement for inclusion in the BRFSS public-use data set.

** The 74 activities were organized into major headings using a modified version of the 2011 Compendium of Physical Activities by Ainsworth, et. al. (https://cdn-links.lww.com/permalink/mss/a/mss_43_8_2011_06_13_ainsworth_202093_sdc1.pdf). Activities were grouped on the basis of similarity and on response rates, with activities having <400 respondents combined into “Other” categories corresponding to the major headings.

**^††^** Some calisthenics activities might be classified as aerobic conditioning exercise.

^§§^ Home activities included household activities (e.g., vacuuming, dusting, or home repair), child care, carpentry, and painting or wallpapering.

**TABLE 2:** Age-specific and age-adjusted* percentages of reporting walking, gardening, or weightlifting as a first or second most frequent activity^†^ among adults with arthritis^§^ who reported engaging in physical activity in the past month,^¶^ by selected characteristics — Behavioral Risk Factor Surveillance System, United States,** 2019

Characteristic	No. of adults with arthritis engaging in physical activity	Age-adjusted % (95% CI)*		
		Walking	Gardening	Weightlifting
**Overall**	**87,299**	**70.0 (69.3–70.7)**	**10.7 (10.3–11.2)**	**10.3 (9.8–10.9)**
**Sociodemographic characteristic**				
**Age group, yrs (unadjusted)**				
18–44	8,107	63.7 (61.8–65.5)	7.0 (6.1–8.0)	12.3 (11.1–13.6)
45–64	30,635	73.5 (72.6–74.5)	12.8 (12.0–13.7)	7.0 (6.5–7.6)
≥65	48,557	71.2 (70.3–72.0)	16.4 (15.7–17.1)	5.5 (5.1–6.0)
**Sex**				
Male	34,886	63.9 (62.9–64.9)	10.9 (10.2–11.6)	10.9 (10.2−11.5)
Female	52,413	76.0 (75.2–76.7)	15.1 (14.5–15.8)	4.7 (4.3−5.1)
**Race/Ethnicity**				
White, non-Hispanic	72,415	70.0 (69.4–70.7)	14.4 (13.9–14.9)	7.3 (6.9−7.7)
Black, non-Hispanic	5,607	75.4 (73.3–77.4)	7.8 (6.7–9.1)	7.9 (6.6−9.4)
Hispanic	3,059	72.8 (69.7–75.7)	11.7 (8.9–15.2)	7.3 (5.8−9.2)
Asian, non-Hispanic	794	72.1 (65.2–78.0)	11.4 (7.4–17.1)	8.7 (5.7−13.1)
American Indian or Alaska Native, non-Hispanic	1,290	74.8 (68.6–80.2)	8.0 (5.4–11.8)	4.4 (3.1−6.3)
Other/Multiple race, non-Hispanic	2,495	68.3 (64.5–71.9)	14.3 (11.7–17.3)	6.0 (4.6−7.9)
**Highest level of education**				
Less than high school graduate	4,963	76.7 (74.5–78.7)	10.5 (9.1–12.0)	3.2 (2.4−4.3)
High school graduate or equivalent	21,782	71.7 (70.4–72.8)	13.6 (12.6–14.6)	5.5 (4.9−6.2)
Technical school or some college	26,276	70.8 (69.6–71.9)	14.5 (13.7–15.4)	6.7 (6.1−7.4)
College degree or higher	34,120	68.1 (67.1–69.1)	12.7 (12.0–13.5)	11.2 (10.5−11.9)
**Employment status**				
Employed or self-employed	30,192	67.7 (66.6–68.8)	13.0 (12.1–13.9)	9.2 (8.6−9.9)
Unemployed	2,822	74.8 (71.2–78.1)	11.6 (9.5–14.1)	5.8 (4.2−8.1)
Retired	41,668	71.0 (69.8–72.2)	14.2 (13.3–15.1)	6.7 (6.0−7.6)
Unable to work or disabled	8,058	79.0 (77.1–80.7)	11.1 (9.9–12.5)	2.1 (1.7−2.7)
Student or homemaker	4,206	73.5 (70.8–76.0)	14.6 (12.7–16.7)	7.1 (5.6−9.1)
**Federal poverty level^††^**				
≤125% FPL	11,478	77.3 (75.7–78.8)	11.0 (10.0–12.2)	3.4 (2.8−4.1)
>125% to ≤200% FPL	12,531	72.8 (71.2–74.3)	13.4 (12.2–14.7)	5.5 (4.6−6.4)
>200% to ≤400% FPL	21,874	70.7 (69.4–71.9)	14.7 (13.8–15.7)	7.2 (6.5−7.9)
>400% FPL	26,569	66.7 (65.5–67.8)	13.3 (12.4–14.2)	11.2 (10.4−12.0)
**Sexual orientation^§§^**				
Straight	48,499	70.6 (69.7–71.4)	13.9 (13.3–14.6)	7.0 (6.5−7.5)
Lesbian, gay, bisexual, queer, or questioning	2,700	74.0 (70.9–76.9)	12.1 (9.9–14.8)	6.6 (4.9−8.8)
**Urban-rural status** ^¶¶^				
Large central metro	11,279	72.4 (70.8–73.9)	11.8 (10.6–13.2)	8.5 (7.6–9.4)
Large fringe metro	15,941	67.9 (66.6–69.2)	12.9 (12.1–13.8)	8.2 (7.4–9.1)
Medium metro	18,392	70.3 (69.1–71.4)	13.4 (12.6–14.3)	7.0 (6.4–7.6)
Small metro	12,587	70.2 (68.7–71.7)	13.9 (12.8–15.1)	6.8 (6.0–7.7)
Micropolitan	14,468	69.6 (68.2–71.1)	14.5 (13.5–15.6)	5.6 (4.9–6.5)
Noncore	14,632	71.9 (70.3–73.5)	15.7 (14.4–17.0)	4.0 (3.3–4.7)
**Health-related characteristic**				
Joint pain severity***				
None/Mild	46,371	69.1 (68.2–70.0)	13.5 (12.8–14.2)	9.4 (8.8−10.0)
Moderate	20,280	71.6 (70.3–72.8)	13.5 (12.6–14.4)	6.5 (5.8−7.3)
Severe	19,421	73.6 (72.4–74.9)	12.7 (11.8–13.7)	4.3 (3.7−4.9)
**Body mass index (kg/m^2^)**				
Underweight or healthy weight (<25)	22,816	68.5 (67.2–69.7)	13.5 (12.6–14.5)	7.9 (7.2−8.7)
Overweight (25 to <30)	30,115	69.1 (68.0–70.1)	13.7 (12.8–14.6)	8.9 (8.3−9.7)
Obese (≥30)	30,171	73.6 (72.6–74.5)	12.9 (12.1–13.6)	5.9 (5.3−6.4)
**Mobility limitations^†††^**				
No	63,303	69.7 (68.9–70.4)	13.9 (13.3–14.5)	8.6 (8.1−9.0)
Yes	23,530	73.9 (72.8–75.1)	11.8 (10.9–12.7)	3.9 (3.3−4.4)
**Arthritis-attributable activity limitations^§§§^**				
No	54,910	70.1 (69.3–70.9)	13.3 (12.7–13.9)	8.6 (8.1−9.1)
Yes	31,562	71.9 (70.9–72.9)	13.4 (12.6–14.1)	5.3 (4.9−5.8)
**Arthritis-attributable work limitations^¶¶¶^**				
No	63,083	70.1 (69.3–70.8)	13.0 (12.5–13.6)	8.7 (8.3−9.3)
Yes	22,660	72.4 (71.3–73.6)	14.0 (13.1–15.0)	4.5 (4.0−5.0)
**Self-rated health**				
Excellent or very good	35,055	67.5 (66.4–68.4)	13.2 (12.5–14.0)	10.5 (9.8−11.2)
Good	31,206	72.1 (71.1–73.1)	14.5 (13.6–15.4)	6.2 (5.7−6.8)
Fair or poor	20,858	74.1 (72.9–75.3)	11.8 (11.0–12.7)	4.2 (3.6−4.8)

**Abbreviations:** CI = confidence interval; FPL = federal poverty level.

* Except for age groups, age-adjusted estimates were generated in weighted logistic regression models that included age as a categorical covariate (18−44 years, 45−64 years, and ≥65 years).

^†^ Those who were engaging in physical activity were classified as participating in an activity if they reported this activity for one of two questions: 1) “What type of physical activity or exercise did you spend the most time doing during the past month?” or 2) “What other type of physical activity gave you the next most exercise during the past month?” Participants who reported one activity but had missing data for the second most frequent activity (e.g., “don’t know” or “refused”) were included in the analysis.

^§^ Respondents were classified as having arthritis if they responded “yes” to the question, “Have you ever been told by a doctor or other health care professional that you have arthritis, rheumatoid arthritis, gout, lupus, or fibromyalgia?”

^¶^ Respondents with arthritis were classified as engaging in physical activity if they responded “yes” to the question, “During the past month, other than your regular job, did you participate in any physical activities or exercises such as running, calisthenics, golf, gardening, or walking for exercise?”

** In 2019, New Jersey did not collect enough data to meet the minimum requirement for inclusion in the BRFSS public-use data set.

^††^ FPL is the ratio of total family income to federal poverty level per family size. Overall, 14,847 adults with arthritis engaging in physical activity had missing FPL data.

**^§§^** Sexual orientation was asked in 30 states (Alaska, Arizona, Colorado, Connecticut, Delaware, Florida, Georgia, Hawaii, Idaho, Iowa, Kansas, Louisiana, Maryland, Minnesota, Mississippi, Montana, New York, North Carolina, Ohio, Oklahoma, Rhode Island, South Carolina, Tennessee, Texas, Utah, Vermont, Virginia, Washington, West Virginia, and Wisconsin). A total of 788 adults with arthritis who engaged in physical activity refused to answer.

^¶¶^ Urban-rural status was categorized using the National Center for Health Statistics 2013 Urban-Rural Classification Scheme for Counties. https://www.cdc.gov/nchs/data/series/sr_02/sr02_166.pdf

*** For the question, “On a scale of 0 to 10 where 0 is no pain or aching and 10 is pain or aching as bad as it can be, during the past 30 days, how bad was your joint pain on average,” an answer of 0−4 was defined as none/mild, an answer of 5−6 was defined as moderate, and an answer of 7−10 was defined as severe.

^†††^ Respondents were classified as having mobility limitations if they responded “yes” to the question, “Do you have serious difficulty walking or climbing stairs?”

^§§§^ Respondents were classified as having arthritis-attributable activity limitations if they responded “yes” to the question, “Are you now limited in any way in any of your usual activities because of arthritis or joint symptoms?”

^¶¶¶^ Respondents were classified as having arthritis-attributable work limitations if they responded “yes” to the question, “In this next question, we are referring to work for pay. Do arthritis or joint symptoms now affect whether you work, the type of work you do, or the amount of work you do?”

The percentage of adults with arthritis who reported gardening increased with age from 7.0% among adults aged 18–44 years to 16.4% among those aged ≥65 years. The age-adjusted prevalence of gardening was higher among women (15.1%) than among men (10.9%), and higher among non-Hispanic White adults (14.4%) than among non-Hispanic American Indian/Alaska Native adults (8.0%) and non-Hispanic Black adults (7.8%). The percentage reporting gardening was lower among those without a high school diploma (10.5%) than among persons with higher levels of educational attainment (12.7%–14.5%). Gardening prevalence increased with increasing rurality.

The prevalence of weightlifting was highest among those aged 18–44 years (12.3%), declined with age, and was higher among men (10.9%) than among women (4.7%) and higher among those who were employed or self-employed (9.2%) than among those who were unable to work or disabled (2.1%). Weightlifting prevalence increased with increasing education, income, and self-rated health and decreased with increasing joint pain severity and rurality.

The median state-specific unadjusted percentage of adults with arthritis who reported walking was 70.5% (range = 62.9% [Hawaii] to 75.4% [Alabama]) (Table 3). The median percentage who reported gardening was 12.6% (range = 3.8% [DC] to 17.6% [Florida], and the median who reported weightlifting was 7.1% (range = 3.6% [Maine] to 13.9% [DC]).

**TABLE 3:** Unadjusted reported prevalence of walking, gardening, or weightlifting as a first or second most frequent activity* among adults with arthritis^†^ who reported engaging in physical activity in the past month^§^ — Behavioral Risk Factor Surveillance System, United States,^¶^ 2019

Jurisdiction	Walking		Gardening		Weightlifting	
	Weighted no.**	Unadjusted % (95% CI)	Weighted no.**	Unadjusted % (95% CI)	Weighted no.**	Unadjusted % (95% CI)
Alabama	548,000	75.4 (72.6−78.0)	111,000	15.3 (13.3−17.6)	39,000	5.3 (4.0−7.1)
Alaska	60,000	74.8 (69.7−79.3)	7,000	9.0 (6.2−12.9)	5,000	5.9 (4.0−8.8)
Arizona	603,000	73.3 (69.9−76.4)	82,000	9.9 (8.2−12.0)	73,000	8.9 (6.9−11.5)
Arkansas	278,000	71.6 (67.8−75.0)	62,000	16.0 (13.6−18.8)	19,000	5.0 (3.4−7.2)
California	3,053,000	74.2 (71.3−76.8)	653,000	15.9 (13.8−18.2)	330,000	8.0 (6.6−9.8)
Colorado	489,000	67.7 (65.1−70.3)	59,000	8.2 (6.8−9.8)	80,000	11.0 (9.4−12.9)
Connecticut	300,000	70.2 (67.3−72.9)	55,000	13.0 (11.2−15.0)	30,000	6.9 (5.5−8.7)
Delaware	86,000	70.4 (65.4−74.9)	16,000	13.0 (10.3−16.3)	7,000	5.3 (3.9−7.4)
District of Columbia	42,000	70.5 (64.8−75.6)	2,000	3.8 (2.3−6.2)	8,000	13.9 (9.7−19.7)
Florida	1,867,000	68.9 (65.7−72.0)	477,000	17.6 (14.5−21.2)	182,000	6.7 (5.2−8.7)
Georgia	793,000	70.2 (66.2−73.9)	137,000	12.2 (9.8−15.0)	96,000	8.5 (5.9−12.1)
Hawaii	100,000	62.9 (59.3−66.4)	24,000	15.3 (13.0−17.9)	12,000	7.3 (5.5−9.5)
Idaho	141,000	63.3 (58.4−67.9)	37,000	16.7 (13.7−20.2)	13,000	5.8 (3.8−8.7)
Illinois	1,067,000	67.6 (64.2−70.9)	209,000	13.2 (11.1−15.7)	130,000	8.3 (6.5−10.5)
Indiana	562,000	73.0 (70.2−75.6)	80,000	10.4 (8.7−12.3)	55,000	7.1 (5.6−8.9)
Iowa	276,000	68.9 (66.4−71.2)	46,000	11.5 (10.0−13.1)	27,000	6.8 (5.6−8.3)
Kansas	257,000	73.3 (70.9−75.5)	43,000	12.4 (10.9−14.1)	25,000	7.2 (5.8−8.8)
Kentucky	460,000	71.8 (68.4−75.0)	89,000	13.8 (11.7−16.3)	38,000	5.9 (4.3−8.2)
Louisiana	399,000	72.5 (68.6−76.1)	88,000	15.9 (13.3−19.0)	35,000	6.4 (4.4−9.3)
Maine	141,000	68.4 (65.5−71.1)	33,000	15.9 (13.9−18.0)	7,000	3.6 (2.5−5.2)
Maryland	522,000	71.5 (69.4−73.6)	81,000	11.1 (9.9−12.5)	62,000	8.6 (7.2−10.1)
Massachusetts	593,000	68.6 (65.3−71.7)	109,000	12.6 (10.5−15.0)	52,000	6.0 (4.6−7.7)
Michigan	1,132,000	73.4 (71.0−75.6)	152,000	9.8 (8.4−11.4)	111,000	7.2 (5.9−8.7)
Minnesota	469,000	71.1 (69.0−73.0)	103,000	15.6 (14.1−17.2)	40,000	6.1 (5.1−7.3)
Mississippi	243,000	73.7 (69.4−77.6)	43,000	12.9 (10.5−15.7)	18,000	5.6 (3.9−7.8)
Missouri	527,000	67.4 (64.0−70.6)	69,000	8.8 (7.1−10.9)	43,000	5.5 (4.3−7.1)
Montana	119,000	68.2 (65.4−71.0)	22,000	12.6 (10.8−14.8)	14,000	8.2 (6.6−10.1)
Nebraska	155,000	72.6 (70.3−74.8)	23,000	10.9 (9.5−12.5)	15,000	7.2 (5.8−8.8)
Nevada	251,000	68.8 (62.0−74.8)	27,000	7.5 (5.3−10.7)	36,000	9.8 (6.3−14.8)
New Hampshire	136,000	71.6 (68.2−74.7)	24,000	12.5 (10.5−14.8)	12,000	6.2 (4.5−8.3)
New Mexico	204,000	73.6 (70.2−76.7)	29,000	10.5 (8.6−12.8)	26,000	9.2 (7.3−11.6)
New York	1,509,000	73.5 (70.9−76.0)	202,000	9.8 (8.4−11.5)	148,000	7.2 (5.8−8.9)
North Carolina	970,000	69.0 (65.1−72.7)	242,000	17.2 (14.4−20.5)	97,000	6.9 (5.2−9.0)
North Dakota	64,000	65.1 (61.1−68.9)	10,000	10.2 (8.2−12.5)	9,000	9.6 (7.4−12.4)
Ohio	1,123,000	68.8 (66.3−71.3)	177,000	10.8 (9.6−12.3)	107,000	6.5 (5.2−8.2)
Oklahoma	325,000	71.1 (67.6−74.3)	42,000	9.1 (7.5−11.1)	35,000	7.6 (5.8−9.9)
Oregon	398,000	65.5 (62.1−68.8)	102,000	16.8 (14.3−19.8)	40,000	6.6 (4.9−8.8)
Pennsylvania	1,277,000	67.2 (64.0−70.3)	241,000	12.7 (10.7−14.9)	164,000	8.6 (6.8−10.9)
Rhode Island	101,000	71.2 (67.8−74.4)	19,000	13.3 (11.2−15.7)	10,000	7.0 (5.2−9.4)
South Carolina	504,000	75.4 (72.6−78.0)	99,000	14.8 (12.7−17.3)	48,000	7.2 (5.6−9.1)
South Dakota	68,000	65.3 (59.1−71.0)	8,000	8.1 (5.7−11.3)	9,000	8.8 (6.0−12.6)
Tennessee	662,000	74.3 (71.0−77.3)	117,000	13.1 (11.0−15.7)	64,000	7.1 (5.4−9.5)
Texas	1,880,000	70.2 (66.4−73.7)	386,000	14.4 (11.9−17.3)	215,000	8.0 (6.1−10.6)
Utah	264,000	67.4 (65.1−69.7)	42,000	10.8 (9.4−12.4)	36,000	9.3 (7.9−10.8)
Vermont	64,000	72.0 (68.8−75.1)	11,000	12.8 (10.8−15.0)	5,000	5.5 (3.9−7.5)
Virginia	765,000	70.0 (67.3−72.7)	130,000	11.9 (10.2−13.8)	68,000	6.3 (5.1−7.7)
Washington	739,000	71.1 (68.9−73.2)	177,000	17.1 (15.4−18.9)	65,000	6.2 (5.3−7.4)
West Virginia	237,000	68.4 (65.3−71.3)	35,000	10.1 (8.3−12.2)	18,000	5.3 (4.0−7.0)
Wisconsin	605,000	74.2 (70.8−77.3)	123,000	15.0 (12.6−17.8)	54,000	6.6 (5.0−8.8)
Wyoming	51,000	70.0 (65.8−73.8)	8,000	11.4 (9.0−14.4)	5,000	7.3 (5.3−10.2)
**Median (49 states and District of Columbia)**	—	70.5	—	12.6	—	7.1
**Guam**	6,000	57.8 (47.2−67.8)	2,000	25.2 (15.5−38.3)	1,000	11.9 (7.4−18.8)
**Puerto Rico**	153,000	68.3 (63.5−72.8)	22,000	9.8 (7.3−13.0)	3,000	—**^††^**

**Abbreviation:** CI = confidence interval.

* Adults engaging in physical activity were classified as participating in an activity if they reported this activity for one of two questions: 1) “What type of physical activity or exercise did you spend the most time doing during the past month?” or 2) “What other type of physical activity gave you the next most exercise during the past month?” Participants who reported one activity but had missing data for the other most frequent activity (e.g., “don’t know,” or “refused”) were included in the analysis.

^†^ Respondents were classified as having arthritis if they responded “yes” to the question, “Have you ever been told by a doctor or other health care provider that you have arthritis, rheumatoid arthritis, gout, lupus, or fibromyalgia?”

^§^ Respondents with arthritis were classified as engaging in physical activity if they responded “yes” to the question, “During the past month, other than your regular job, did you participate in any physical activities or exercises such as running, calisthenics, golf, gardening, or walking for exercise?”

^¶^ In 2019, New Jersey did not collect enough data to meet the minimum requirement for inclusion in the BRFSS public-use data set.

** Weighted number represents the estimated number of adults with arthritis engaging in physical activity who reported the activity (walking, gardening, or weightlifting) as their first or second most frequent activity.

^††^ Unreliable estimate (relative standard error >30%).

## Discussion

In 2019, walking was overwhelmingly the most common activity among adults with arthritis who engaged in nonwork–related physical activity in the past month, followed by gardening and weightlifting. The most common activities in this report parallel the activities for adults with mobility disabilities, whose most common activities in 2017 were walking and gardening (*3*). These similarities are expected because arthritis is a leading cause of disability (*4*). Despite arthritis being a cause of pain and disability, walking prevalence increased with increasing joint pain severity. A previous report on walking using national data described a similar finding, specifically for lower extremity joint pain (*5*). Collectively, these findings might signify that the presence of pain might not automatically preclude walking, other physical activities, and their associated benefits.

Walking is an ideal activity for adults with arthritis because it can be inexpensive, safe, convenient, low-impact, and adaptable to individual fitness levels.*** The American College of Rheumatology and the Arthritis Foundation recommend that health care providers offer specific guidance to patients with arthritis regarding physical activity (*6*). This report identifies activities to which adults with arthritis seem amenable. These findings could help health care providers encourage patients to participate in these common activities, including referring them to low-cost physical activity programs delivered by worksites and community organizations.

The cost of physical activity is an important consideration for adults with arthritis (*7*). Whereas all adults with arthritis can benefit from physical activity, those with the lowest levels of household income are more likely to be inactive (*8*). In this report of adults who engaged in physical activity, type of physical activity varied by income level. For example, adults with lower socioeconomic status had lower weightlifting and higher walking prevalences compared with those with higher incomes. Adults with arthritis who are inactive and have lower incomes might be more receptive to low-cost physical activities, such as walking (*7*).^†††^

Adults with arthritis experience optimal health benefits through diverse physical activity regimens, including aerobic, muscle strengthening, and balance exercises (*1*). Benefits of gardening include reduced stress and fatigue and improved mental health and quality of life (*9*). Muscle strengthening can improve fitness and independence, prevent muscle loss, and reduce arthritis pain (*1*). Low-cost muscle strengthening activity options, including lifting objects (e.g., dumbbells, cans of food, or water bottles), using resistance bands, and engaging in bodyweight exercises, are all suitable activities for adults with arthritis.^§§§^

The findings in this report are subject to at least six limitations. First, BRFSS data are self-reported, which can introduce recall and social desirability biases and potential misclassification of activities. Second, the relatively low state-specific response rates (as low as 37.3%) might reduce generalizability and bias the findings. Third, specific activity participation might be underestimated because only the two most frequent activities per person could be reported and data were assessed only for leisure-time (nonwork) activities. Fourth, differences in other activities by characteristics such as income were not assessed. Fifth, data was available for only 49 states and aggregated data might not be nationally representative. Finally, this study provides estimates of reported activities undertaken versus preferred; health care providers might find that this affects physical activity sustainability among patients.

To promote physical activity among adults with arthritis, health care providers can offer advice or counseling for walking or referrals to low-cost, evidence-based physical activity programs.^§§§^ These programs might help adults with arthritis overcome common barriers to physical activity, including cost, lack of instructions about preventing risk for injury while exercising, and fear of arthritis worsening (*7*). Communities can address physical environment barriers to walking by providing safe and supportive infrastructures such as sidewalks, benches, and green spaces.^¶¶¶^ Promoting engagement in physical activity among adults with arthritis can reduce their risk for chronic health conditions and improve their mental health and quality of life.

**Summary**

**What is already known about this topic?**

Among adults with arthritis, physical activity can reduce pain, disability, and functional limitations, and improve mental health and quality of life; however, just over one third of adults with arthritis are aerobically active.

**What is added by this report?**

Approximately 71% of adults with arthritis who engaged in physical activity in the past month reported walking as one of their two most frequent activities. Gardening (13.3%) and weightlifting (7.3%) were the second and third most common activities.

**What are the implications for public health practice?**

Health care providers can help inactive adults with arthritis become active and, by encouraging physical activity and referring them to evidence-based physical activity programs, improve their health and quality of life.

## Footnotes

* The earlier study used the 2008 Physical Activity Guidelines for Americans (https://health.gov/sites/default/files/2019-09/paguide.pdf), which are equivalent to the most recent (2018) Physical Activity Guidelines for Americans, 2nd Ed. https://health.gov/sites/default/files/2019-09/Physical_Activity_Guidelines_2nd_edition.pdf

†
https://www.cdc.gov/brfss/about/index.htm

§ This analysis included 49 states and the District of Columbia. In 2019, New Jersey did not collect enough data to meet the minimum requirement for inclusion in the BRFSS public-use data set.

¶
https://www.cdc.gov/brfss/annual_data/2019/pdf/2019-sdqr-508.pdf

**
https://www.cdc.gov/arthritis/basics/types.html

†† A specific activity was counted once if it was reported in response to one of the following questions: “What type of physical activity or exercise did you spend the most time doing during the past month?” or “What other type of physical activity gave you the next most exercise during the past month?” Participants who reported one activity but had missing data for the second most frequent activity (e.g., “don’t know” or “refused”) were included in the analysis. Among 87,299 adults with arthritis engaging in physical activity, 77,733 participants answered at least the first question (7,859 reported “Don’t know,” 366 refused, and 1,341 responses were missing). The 74 activities were organized into major headings using a modified version of the 2011 Compendium of Physical Activities by Ainsworth et al. (https://cdn-links.lww.com/permalink/mss/a/mss_43_8_2011_06_13_ainsworth_202093_sdc1.pdf). Activities were grouped on the basis of similarity and on response rates, with activities having <400 respondents combined into “Other” categories corresponding to the major headings.

§§ Age-adjusted estimates were generated in weighted logistic regression models that included age as a categorical covariate (18−44 years, 45−64 years, and ≥65 years).

¶¶ 45 C.F.R. part 46.102(l)(2), 21 C.F.R. part 56; 42 U.S.C. Sect. 241(d); 5 U.S.C. Sect. 552a; 44 U.S.C. Sect. 3501 et seq.

***
https://www.hhs.gov/sites/default/files/call-to-action-walking-and-walkable-communites.pdf

†††
https://www.cdc.gov/arthritis/basics/physical-activity-overview.html

§§§ https://www.arthritis.org; https://www.cdc.gov/arthritis/interventions/physical-activity.html

¶¶¶
https://www.thecommunityguide.org/findings/physical-activity-built-environment-approaches
